# Metabolomics Reveals the Alteration of Metabolic Pathway by Alpha-Melanocyte-Stimulating Hormone in B16F10 Melanoma Cells

**DOI:** 10.3390/molecules25153384

**Published:** 2020-07-26

**Authors:** Seung-Ho Seo, Jae Kwon Jo, Eun-Ju Kim, Seong-Eun Park, Seo Yeon Shin, Kyung Mok Park, Hong-Seok Son

**Affiliations:** 1 School of Korean Medicine, Dongshin University, Naju, Jeonnam 58245, Korea; blue784300@naver.com (S.-H.S.); jojk89@naver.com (J.K.J.); yci3431@naver.com (E.-J.K.); seong9525@naver.com (S.-E.P.); 2 Department of Pharmaceutical Engineering, Dongshin University, Naju, Jeonnam 58245, Korea; ssy33144@naver.com

**Keywords:** α-melanocyte-stimulating hormone, melanoma cell, metabolomics, energy metabolism, amino acid metabolism

## Abstract

The purpose of this study was to understand the changes of metabolic pathway induced by alpha-melanocyte-stimulating hormone (α-MSH) in B16F10 melanoma cells in an untargeted metabolomics approach. Cells were treated with 100 nM of α-MSH and then incubated for 48 h. α-MSH increased tyrosinase activity and melanin content by 56.5 and 61.7%, respectively, compared to untreated cells after 48 h of cultivation. The clear separation between groups was observed in the principal component analysis score plot, indicating that the levels of metabolites of melanoma cells were altered by treatment with α-MSH. Metabolic pathways affected by α-MSH were involved in some amino acid metabolisms. The increased levels of fumaric acid, malic acid, oxaloacetic acid and citric acid related to the citric acid cycle pathway after α-MSH treatment suggested enhanced energy metabolism. Metabolic pathways altered by α-MSH treatment can provide useful information to develop new skin pigmentation inhibitors or anti-obesity drugs.

## 1. Introduction

Melanogenesis can be stimulated by the ultraviolet or visible light-induced alpha-melanocyte-stimulating hormone (α-MSH) in melanocytes [1]. While α-MSH, an endogenous peptide hormone and neuropeptide of the melanocortin family, is well known for its physiological function of stimulation of melanin production, it also plays an important role in controlling appetite and energy balance [2], sexual activity [3] and ischemia and reperfusion-associated injury [4]. The effects of α-MSH to protect ischemic damage in various organs, such as brain [5] and gastrointestinal tract [6], have been reported. In addition, several studies have shown that α-MSH can reduce UV-induced skin damage through inositol trisphosphate (IP_3_) kinase-Akt pathway [7] and nucleotide excision repair [8]. Potential strategies have been proposed to prevent skin cancer by improving pigmentation with α-MSH analogs [9] or cAMP agonists [10]. However, an important concern about α-MSH is its overproduction causing various skin diseases, such as chloasma, freckles and skin cancer [11]. Therefore, in order to investigate the potential efficacy and side effects of α-MSH, a comprehensive analysis of α-MSH on the metabolic pathway of cells is required.

Metabolomics is a very powerful tool that provide a more comprehensive understanding of the effects of biological potential at a specified time in particular environmental conditions [12]. With the development in the last few decades of analytical instruments, such as mass spectrometry and nuclear magnetic resonance spectroscopy, metabolomic analysis of cells has been widely used to perform a complete assessment of functional cellular responses to pathogen infections, toxicity and environmental factors [13]. In addition, studies have reported that metabolites directly initiate cell signaling stages and regulate various biological processes such as post-translational modifications and epigenetic mechanisms [14]. Thus, metabolomic analysis is expected to provide novel insight into the effects of α-MSH.

Melanogenesis by α-MSH is a complex process that is controlled by tyrosinase and related proteins. The α-MSH binds to melanocortin-1 receptor (MC1R) in melanocytes to activate adenylate cyclase, which leads to an elevated level of intracellular cAMP. The expression of tyrosinase is stimulated by cAMP pathway [15]. Tyrosinase mediates hydroxylation of tyrosine into DOPA, followed by a series of processes to synthesize melanin [16]. Therefore, inhibition of tyrosinase has been widely used in the industry to reduce excessive pigmentation [1]. However, as noted, the series of successive melanogenesis pathways do not capture the full range of behaviors of a metabolic network. As metabolic networks generally include various branch points, α-MSH can cause alteration of unknown metabolic pathways. However, little effort has been devoted to the metabolic profiling of melanogenesis in melanoma cells. Herein, we applied this strategy to better understand the changes of metabolic pathway induced by α-MSH in B16F10 melanoma cells in an untargeted approach.

## 2. Results

### 2.1. Effect of α-MSH on Tyrosinase Activity and Melanin Synthesis

To investigate the effect of α-MSH, tyrosinase activity and melanin production in B16F10 melanoma cells were analyzed. As expected, α-MSH increased tyrosinase activity and melanin content by 56.5% and 61.7%, respectively, compared to untreated cells at 48 h (Figure S1).

### 2.2. Metabolite Changes During Cell Culture Period

**Figure 1:**
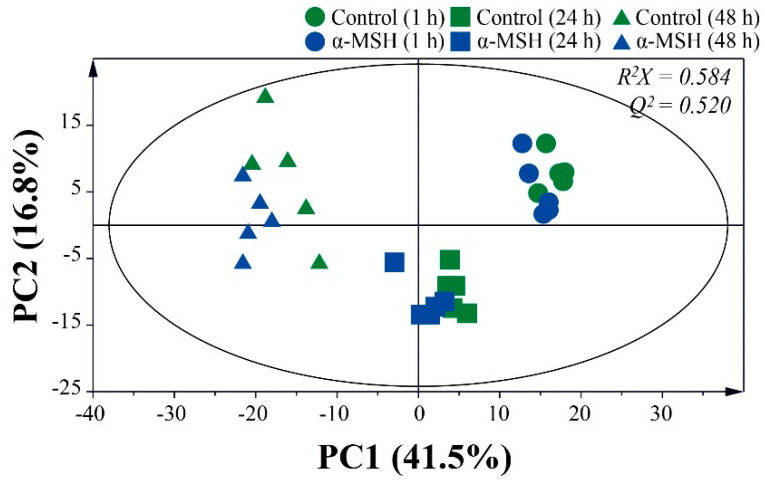
Principal component analysis (PCA) score plot based on the gas chromatography-mass spectrometry (GC-MS) data sets during 48 h of melanoma cell culture.

Principal component analysis (PCA) was performed to monitor changes in metabolites during 48 h of melanoma cell culture (Figure 1). The first and second principal component (PC1 and PC2) represented 41.5% and 16.8% of the total variance, respectively. The clear separation of samples by cultivation time was observed in the PCA score plot, indicating that metabolites change rapidly during the cell culture period. The differences of metabolites by α-MSH were much less than those by the cell culture period. The two groups at 1 h were not fully distinguishable in the PCA score plot, implying that metabolites were similar to each other. However, the PCA score plot at 24 h showed clear differentiation, suggesting that the metabolites of melanoma cells can be altered by treatment with α-MSH. Based on the fragmentation pattern of National Institute of Standards and Technology (NIST) library and in-house library made by standard chemicals, a total of 52 metabolites were identified in the sample (Table S1). Most metabolites, except malic acid, glutamine, glutamic acid and aspartic acid, increased significantly during the cell culture period (Table S2). Melanoma cells treated with α-MSH showed similar metabolite changes but a significant decrease in the content of glucose was observed (Table S3).

### 2.3. Effect of α-MSH on Metabolic Changes After 48 h of Cell Culture

**Figure 2:**
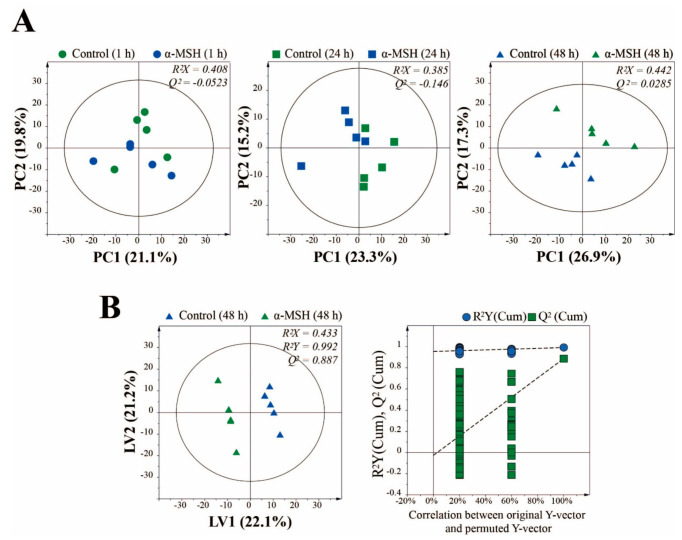
(**A**) PCA score plots derived from GC-MS data at 1, 24 and 48 h of melanoma cell culture. (**B**) partial least squares-discriminant analysis (PLS-DA) score plot between control and α-MSH treated group after 48 h of incubation validated by a permutation test.

PCA was performed to investigate the metabolic differences between control and α-MSH during cell culture period (Figure 2A). The two groups at 1 and 24 h of cultivation were not completely distinguished in the PCA score plot, indicating that the metabolite profiles between the groups were not significantly different. However, after 48 h of cell culture, separation between the groups by PC2 was observed, suggesting that the metabolites of melanoma cells can be altered by treatment with α-MSH. To maximize separation and find potential biomarkers, partial least squares-discriminant analysis (PLS-DA) model for supervised pattern recognition was further applied between the experimental groups. The PLS-DA score plot (Figure 2B) showed that the two groups were clearly separated from each other, indicating that there was a significant metabolite difference between the two groups. The permutation test supported the validity of this PLS-DA model. To find the metabolite responsible for the separation of this PLS-DA model, the variables of variable importance in projection (VIP) were determined. Potential metabolic biomarkers were selected based on VIP > 1.0 and the corrected *p* values. The α-MSH treated melanoma cells were characterized by higher levels of citric acid, glycine, oxaloacetic acid, malic acid, lactic acid, glycolic acid, fumaric acid and glutamine but lower levels of inositol, compared to the control (Table 1). Interestingly, the changes in levels of malic acid, inositol and glutamine showed opposite results to the change pattern according to the cell culture period. The levels of fumaric acid did not change significantly with cell culture but α-MSH-treated samples showed significantly higher levels than control. It is presumed that these metabolites are related to the metabolic pathway that changes dramatically by α-MSH treatment.

### 2.4. Effect of α-MSH on Metabolic Pathway Alteration

**Figure 3:**
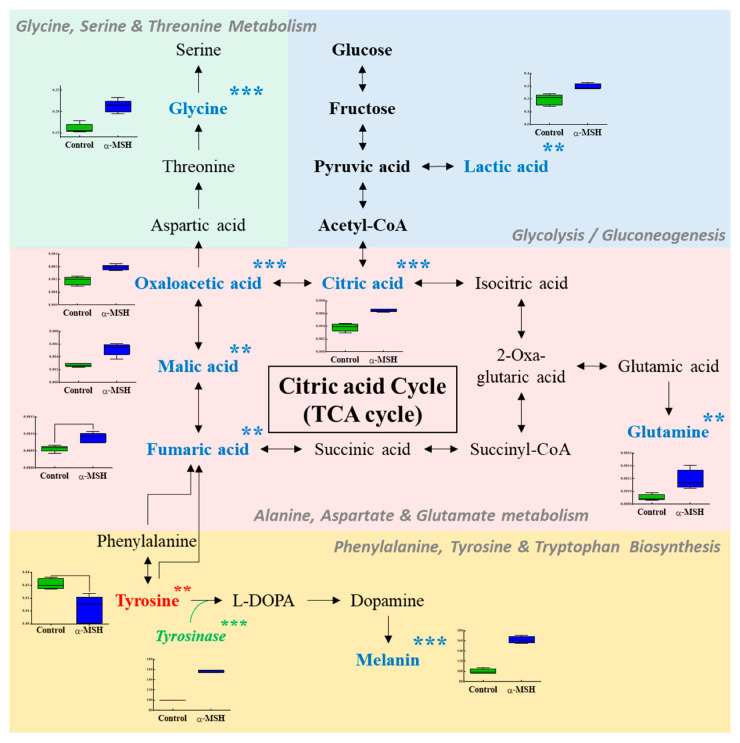
Schematic of the metabolic pathway related to the effects of α-MSH. The metabolites in blue and red represent the potential biomarkers that are increased or decreased by α-MSH treatment, respectively. Symbols (*) indicate significant difference (* *p* < 0.05; ** *p* < 0.01; *** *p* < 0.001).

Metabolic pathway analysis was performed to identify relevant metabolic pathways affected by α-MSH (Figure 3). This analysis shows metabolic pathways by enrichment analysis and impact values by topology analysis. Eight important pathways were identified, based on the pathway impact and −log(*p*) value (Table 2). Metabolic pathways affected by α-MSH were involved in alanine-aspartate and glutamate metabolism, phenylalanine-tyrosine and tryptophan biosynthesis, glutamine and glutamate metabolism, cysteine and methionine metabolism, glycine-serine and threonine metabolism, citrate cycle, taurine and hypotaurine metabolism and phenylalanine metabolism. Figure 3 shows a schematic of the affected metabolic pathways by α-MSH treatment. In α-MSH treated cells, the content of some metabolites, including tyrosine, fumaric acid, malic acid, oxaloacetic acid, citric acid, lactic acid, glutamine and glycine, was significantly different from that of control cells.

## 3. Discussion

The α-MSH exerts its effect by binding to 5 subtypes of melanocortin receptors (MC1-R to MC5-R), which are known to involve the melanocortins signal, including α-MSH [17]. Activation of different MC-Rs regulates different cellular functions and behaviors in various types of cells. For example, MC4-R contributes to the regulation of food intake, energy consumption and fatty acid oxidation in skeletal muscle [18]. On the other hand, activation of MC1-R mediates melanin production and temperature regulation [19]. While MC1-R was originally proven in melanocyte-derived melanoma cells, there is now also evidence that it is expressed in many types of cells, including immune cells [20]. Therefore, it is important to identify differences in cell metabolism according to the activation of different subtypes of MC-Rs but few studies have been conducted to date. The interaction between α-MSH and MC1-R promotes melanogenesis through the cAMP/PKA pathway. However, there was little interest in the metabolism altered by α-MSH in melanoma cells, except for this pathway. Maresca et al. [21] have discovered a new α-MSH⁄peroxisome proliferator activated receptor-γ (PPARγ) connection that influenced both pigmentation and proliferation in B16-F10 cells. Although α-MSH activity as a mitogenic agent in melanocytes is well known, some studies have shown that the α-MSH⁄PPARγ pathway down-regulated proliferation in melanoma cell lines [22].

Although many studies of melanogenesis by α-MSH in melanoma cells have been published, little attention has been paid to the altered metabolism by α-MSH in melanoma cells. Metabolomics can provide a comprehensive identification of pathway alterations by treatment. To the best of our knowledge, this is the first study to identify metabolite changes in melanoma cells by α-MSH treatment. In this study, an increase in fumaric acid, malic acid, oxaloacetic acid and citric acid related to the citric acid cycle pathway were observed after α-MSH treatment. However, the administration of α-MSH suppressed citric acid synthase in a rodent model [23]. The increase of metabolites of citric acid cycle by α-MSH seems to enhance the energy metabolism. These results may be related to other findings that α-MSH can control body weight through its hypermetabolic and hyperthermic effects [24]. It is well known that the hypothalamic melanocortin system is involved in thermoregulation [25]. Meanwhile, there are some reports that α-MSH is involved in energy balance by regulating appetite stimulation based on the relationship with orexigenic neuropeptides and leptin [26,27]. Kravchychyn et al. [28] reported that the long-term α-MSH treatment promoted a significant improvement in body adiposity through the regulation of anorexigenic/orexigenic pathways in obese adolescents. From the above results, α-MSH appears to be an important substance involved in the complex mechanisms of energy balance control.

The signal pathway of α-MSH associated with hypermetabolic effects can be an attractive candidate drug target for the treatment of obesity. For example, the MC4-R activation plays an important role in weight regulation, which provides insight into the mechanisms that can be used as a new target for obesity [29]. The intracellular mechanism by which α-MSH activates energy metabolism is not well understood but two possible hypotheses from previous studies are possible. First, according to the results of Møller et al. [22], α-MSH stimulates glucose uptake by an unknown mechanism, boosting central effects on metabolism. However, in this study, there was no significant difference in glucose content between the control and α-MSH treated groups. Second, the metabolic pathway of tyrosine degradation ends up as fumaric acid and acetoacetate, which can enter the citric acid cycle [30]. In this study, acetoacetate was not detected but the content of fumaric acid increased significantly with α-MSH treatment. However, there are no reports of α-MSH promoting energy metabolism via glucose uptake or tyrosine degradation. Although increased tyrosinase activity by α-MSH may affect the energy metabolic pathway, further studies are needed to demonstrate this. Since the metabolic network generally contains various branches, changes in the metabolic pathway by α-MSH can then lead to unknown metabolic pathway changes. Hill et al. [31] reported that the accumulation of cAMP induced by α-MSH showed a strong positive correlation with the ability of B16 melanoma clones to form pulmonary tumor colonies, suggesting that it is linked with biochemical pathways that are responsible for the formation of experimental metastasis. In this study, α-MSH treatment did not increase cellular proliferation. Although the hypermetabolic effects by α-MSH may be associated with cancer proliferation and metastasis, there is no report that α-MSH is related to cancer cell proliferation and metastasis. Further studies are needed on the effects of α-MSH on metabolism change of cancer cell.

Although this study focused on the intracellular metabolome, metabolic profiling of medium supernatants could provide complementary information on the metabolites consumed and excreted by cells. However, the culture medium contains specific compounds, including amino acids, vitamins, inorganic salts and glucose, to provide energy sources and regulate the cell cycle [32]. In this study, medium supernatants were not analyzed because compounds present in the culture medium can be mixed with metabolites released from the cells, ultimately obscuring the profile of the secretome. Daskalaki et al. [33] reported that multiple and cell-free medium combinations should be tested to exclude the influence of culture media when designing a cell secretion-based metabolite profiling experiments. Another limitation of the current study is that the data obtained are based on an up-regulated pigmentary model of B16F10 melanoma cells. Tumor cells metabolize differently from normal cells. For example, a tumor cell may have Warburg effect that it depends on the metabolic pathways of glycolysis or gluconeogenesis to meet their metabolic demand [34]. Since melanogenesis in B16F10 melanoma cells is several times up-regulated compared to normal melanocytes, further studies on comparative metabolic analysis using normal melanocytes are needed to confirm our results.

## 4. Materials and Methods 

### 4.1. Cell Culture

The B16F10 melanoma cells (CRL-6475) were purchased from the American Type Culture Collection (ATCC, Manassas, VA, USA). Cells were incubated in Dulbecco’s modified Eagle’s medium (DMEM) with 10% fetal bovine serum and 1% penicillin/streptomycin in air containing 5% CO_2_ at 37 °C. Cells were sub-cultured every 3 days. Cells were seeded in 12-well plates, at a density of 1 × 10^5^ cells/well and incubated for 24 h. α-MSH (100 nM) was added to the wells of the plate and the cells then incubated for an additional 48 h. Through the MTT assay, we determined the concentration (100 nM) that could have effects without affecting the viability of B16F10 cells compared to the untreated control group.

### 4.2. Measurement of Cellular Melanin Contents

Cell pellets were lysed in NaOH (1 N) in 10% DMSO at 80 °C for 1 h. The relative melanin content was determined by measuring absorbance at the 490 nm using a microplate reader as previously described [35].

### 4.3. Measurement of Cellular Tyrosinase Activity

Cells were washed using cold-phosphate-buffered saline (PBS) and lysed with Pro-Prep lysis buffer (iNtRON Biotechnology, Seongnam, Korea). The cell lysate was purified by centrifugation at 12,000 rpm for 15 min at 4 °C. Enzyme activity was normalized to protein concentration (30 µg), as determined by Bradford assay. Cellular tyrosinase and L-DOPA solution (0.1 M sodium phosphate) were evaluated at 37 °C for 1 h. The absorbance at 405 nm was measured by microplate reader.

### 4.4. Sample Preparation

Cells were washed with 0.9% NaCl. Then, 200 µL of distilled water and 200 µL of methanol were added to the cells on ice. Each well of the plate was scraped with a cell scraper. The cell extract was then transferred to an Eppendorf tube containing 200 µL of cold chloroform. Cells were stirred for 20 min on a shaker, then centrifuged at a minimum of 16,100× *g* for 5 min at 4 °C. Polar (upper) phase (100 μL) was transferred to a vial, without touching the interphase. Quality control (QC) samples were prepared by collecting the same volume (10 μL) of polar phase from each sample prior to the drying process. For blank samples, the same amount of water as the sample was added into an Eppendorf tube. After the polar phase was dried on a rotary vacuum evaporator at −4 °C, vials were capped and stored at −80 °C until analysis. 

### 4.5. Sample Derivatization

After freeze drying, 80 μL of *O*-methoxyamine hydrochloride (20 mg/mL) in pyridine solution was added to each sample. After vortex-mixing each sample for 30 s, all samples were incubated at 30 °C for 90 min in the dark. After adding 30 μL of *N*-methyl-*N*-trimethylsilyl-trifluoroacetamide containing 1% trimethylchlorosilane, silylation process was performed. Each sample was vortex mixed for 30 s and then incubated at 37 °C for 30 min. Ten μL of ribitol (0.5 mg/L) was added as an internal standard (IS). After centrifuging the sample at 13,000 rpm for 10 min, GC-MS analysis was performed on the supernatant. 

### 4.6. GC-MS Analysis

Derivatized samples were analyzed using GC-MS (QP2020, Shimadzu, Kyoto, Japan). Rtx-5MS with fused silica capillary column (30 m × 0.25 mm ID, J&W Scientific, CA) was used for the separation of metabolites. The front inlet temperature was 230 °C. The column temperature was maintained at 80 °C for 2 min isothermally and then raised by 15 °C/min to 330 °C and held there for 6 min isothermally. The transfer line and ion source temperatures were 250 and 200 °C, respectively. Ionization was achieved with a 70 eV electron beam. The helium gas flow rate through the column was 1 mL/min. Twenty scans per second were recorded in a mass range of 85–500 *m*/*z*. Chromatograms and mass spectra were obtained using Shimadzu GC solution (Shimadzu, Kyoto, Japan). To measure the stability and performance of the instrument along with the reproducibility of the sample treatment procedure, QC samples were analyzed every 5 samples during the run. QC samples were clustered centrally in the PCA score plot, ensuring the reliability of metabolomics analysis (Figure S2). 

### 4.7. Data Processing and Statistical Analysis

GC-MS data was converted to a netCDF format file and processed with MetAlign software for peak detection and alignment [36]. The MetAlign parameters were set according to the AIoutput scaling requirements—a peak slope factor of 2, peak threshold factor of 4, peak threshold of 10 and average peak width at half height of 25, which corresponds to a retention time of 3–26 min and mass range of 85–500 for GC-MS. The resulting data (CSV-format file) was imported into AIoutput software for peak identification and prediction [37]. Feature intensities were normalized according to the intensity of IS (RT 11.205, *m*/*z* 147) prior to multivariate statistical analyses. The height of each feature was divided by the height of IS detected in that particular sample. PCA and PLS-DA of GC-MS data were performed to visualize the variance of metabolites using SIMCA-P 15.0 (Umetrics, Umea, Sweden). For model validation, 200-fold cross-validation was performed. Metabolites with VIP score greater than 1.0 and *p* value from Students *t*-test lower than 0.05 were considered metabolites that were capable of discriminating groups. To correct for multiple testing, the positive false discovery rate (type 1 error) was used by computing *q*-values after *t*-test. Identification of metabolites was performed by comparing the mass spectrum with AIoutput software, NIST 14.0 library and the human metabolome database (HMDB, http://www.hmdb.ca).

### 4.8. Metabolic Pathway Analysis

Metabolic pathway analysis was performed with MetaboAnalyst web software (metaboanalyst.ca) and the Kyoto Encyclopedia of Genes and Genomes (KEGG), by filtering the dataset using an FDR-adjusted *p* value < 0.05 and impact value > 0.1, to reveal how significant metabolites are involved in different pathways [38].

## 5. Conclusions

Our study indicates that the effects of α-MSH are a rearrangement of citric acid cycle pathways and some amino acid metabolisms through several important metabolite changes. A more in-depth analysis of these mechanisms could provide useful information to develop new skin pigmentation inhibitors or anti-obesity drugs.

## Figures and Tables

**Table 1:** Significantly different metabolites between the control and α-MSH treated groups, after 48 h of incubation of melanoma cells.

No.	Metabolites	VIP Score	α-MSH/ Control (↑/↓) ^1^	*p* Value	*q* Value ^2^	Significance ^3^	Control 48 h / 1 h (↑/↓)
1	Citric acid	2.15	↑	0.0001	0.002	***	↑ ***
2	Glycine	2.02	↑	0.0002	0.005	***	↑ ***
3	Oxaloacetic acid	2.01	↑	0.0002	0.007	***	↑ ***
4	Malic acid	1.97	↑	0.0004	0.010	**	↓ **
5	Inositol	1.94	↓	0.0007	0.012	**	↑ ***
6	Lactic acid	1.93	↑	0.0028	0.014	**	↑ ***
7	Glycolic acid	1.92	↑	0.0025	0.017	**	↑ ***
8	Fumaric acid	1.84	↑	0.0039	0.019	**	-
9	Glutamine	1.81	↑	0.0100	0.021	*	↓ *

^1^ The vertical arrows (↓ and ↑) represent a decrease or increase in metabolite levels after 48 h of incubation. ^2^ False discovery rate (FDR). The false discovery rate at 5% was applied to all tests to correct for multiple testing. ^3^ Symbols (*) indicate significant difference (* *p* < 0.05; ** *p* < 0.01; *** *p* < 0.001).

**Table 2:** Metabolic pathways affected by α-MSH on B16F10 melanoma cell.

No.	Metabolic Pathways	Total	Hits	−log(*p*)	Impact
1	Alanine, aspartate and glutamate metabolism	28	7	10.99	0.63
2	Phenylalanine, tyrosine and tryptophan biosynthesis	4	2	5.14	1.00
3	Glutamine and glutamate metabolism	6	2	4.26	0.50
4	Cysteine and methionine metabolism	33	4	3.93	0.33
5	Glycine, serine and threonine metabolism	33	4	3.93	0.46
6	Citrate cycle (TCA cycle)	20	4	5.82	0.28
7	Taurine and hypotaurine metabolism	8	2	3.68	0.71
8	Phenylalanine metabolism	10	2	3.25	0.36

## Supplementary Materials

The following are available online, **Table S1**: Identified metabolites by GC-MS in melanoma cells, **Table S2**: Significantly changed metabolites in melanoma cells after 48 h of incubation, **Table S3**: Significantly changed metabolites in melanoma cells treated with α-MSH after 48 h of incubation, **Figure S1**: Box plots of tyrosinase activity and melanin content, **Figure S2**: PCA score plot derived from GC–MS data of experimental samples and QC samples.
