# Supplementary material for: Metabolomics Reveals the Alteration of Metabolic Pathway by Alpha-Melanocyte-Stimulating Hormone in B16F10 Melanoma Cells
Source: Molecules. 2020 Jul 26;25(15):3384. doi: 10.3390/molecules25153384 (PMC7436294; doi:10.3390/molecules25153384)
Supplement: Supplementary file 1 [file molecules-25-03384-s001.pdf]

Supplementary Materials

Metabolomics Reveals the Alteration of Metabolic Pathway by Alpha-Melanocyte-Stimulating Hormone in B16F10 Melanoma Cells

Seung-Ho Seo [1,†], Jae Kwon Jo [1,†], Eun-Ju Kim [1], Seong-Eun Park [1], Seo Yeon Shin [2], Kyung Mok Park [2,*] and Hong-Seok Son [1,*]

[1] School of Korean Medicine, Dongshin University, Naju, Jeonnam 58245, Republic of Korea

[2] Department of Pharmaceutical Engineering, Dongshin University, Naju, Jeonnam 58245, Republic of Korea

* Correspondence: parkkm@dsu.ac.kr (K.M.P.), hsson@dsu.ac.kr (H.-S.S.); Tel.: +82-32-551-3629 (K.M.P.), +82-61-330-3513 (H.-S.S.).

† These authors equally contributed to the work.

Table 1. Identified metabolites by GC-MS in melanoma cells.

No.	Metabolites	Retention time (min)	Target Mass	HMDB[1)]	PubChem[2)]	KEGG[3)]
1	Propyleneglycol	4.305	117	HMDB0001881	1030	C00583
2	Oxaloacetica acid	4.87	174	HMDB0000223	970	C00036
3	Glycolic acid	4.985	147	HMDB0000115	757	C03547
4	Lactic acid	4.99	117	HMDB0000190	61503	C00256
5	Ketovaline	6.25	89	HMDB0000019	49	C00141
6	2-Aminoisobutyric acid	6.59	130	HMDB0001906	6119	C03665
7	3-Hydroxybutyric acid	6.675	147	HMDB0000357	441	C01089
8	Malonic acid	6.85	147	HMDB0000691	867	C04025
9	Leucine	7.24	158	HMDB0000687	6106	C00123
10	Glycerol	7.26	147	HMDB0000131	753	C00116
11	Phosphoric acid	7.28	299	HMDB0001429	57424078	C00009
12	Isoleucine	7.465	158	HMDB0000172	6306	C00407
13	Proline	7.515	142	HMDB0000162	145742	C00148
14	Glycine	7.61	174	HMDB0000123	750	C00037
15	Fumaric acid	7.915	245	HMDB0000134	444972	C00122
16	Serine	8.1	204	HMDB0000187	5951	C00065
17	Alanine	8.105	100	HMDB0000161	5950	C00041
18	Threonine	8.36	218	HMDB0000167	6288	C00188
19	3-Methylglutaric acid	8.675	147	HMDB0000752	12284	-
20	Malic acid	9.24	147	HMDB0000744	525	C03668
21	Acetylsalicylic acid	9.475	267	HMDB0001879	2244	C01405
22	Aspartic acid	9.515	232	HMDB0000191	5960	C00049
23	Methionine	9.53	176	HMDB0000696	6137	C00073
24	Pyroglutamic acid	9.58	156	HMDB0000267	7405	C01879
25	Creatinine	9.88	115	HMDB0000562	588	C00791
26	Hypotaurine	10.22	188	HMDB0000965	107812	C00519
27	Glutamic acid	10.305	246	HMDB0000148	33032	C00025
28	Phenylalanine	10.43	218	HMDB0000159	6140	C00079
29	5-Aminovaleric acid	10.485	174	HMDB0003355	138	C00431
30	Taurine	10.78	326	HMDB0000251	1123	C00245
31	Ribose	10.835	103	HMDB0000283	5779	C00121
32	N-Formyl-L-Methionine	10.96	85	HMDB0001015	6995182	C03145
33	Glutamine	11.49	156	HMDB0000641	5961	C00064
34	O-Phosphoethanolamine	11.605	299	HMDB0000224	1015	C00346
35	Citric acid	11.845	147	HMDB0000094	311	C00158
36	Adenine	12.22	264	HMDB0000034	190	C00147
37	Fructose	12.27	103	HMDB0000660	439709	C02336
38	Glucose	12.46	319	HMDB0001151	12285879	C01487
39	Lysine	12.52	174	HMDB0000182	5962	C00047
40	Tyrosine	12.66	218	HMDB0000158	6057	C00082
41	Sorbitol	12.68	319	HMDB0000247	5780	C00794
42	Gallic acid	12.76	281	HMDB0005807	370	C01424
43	Palmitoleic acid	13.24	117	HMDB0003229	5312427	C08362
44	N-a-Acetyl-L-Lysine	13.48	174	HMDB0000446	192590	C12989
45	Inositol	13.735	217	HMDB0000211	-	C00137
46	Heptadecanoic acid	13.845	117	HMDB0002259	10465	-
47	Elaidic acid	14.305	117	HMDB0000573	637517	C01712
48	Cystathionine	14.335	218	HMDB0000099	439258	C02291
49	Tryptophan	14.485	202	HMDB0000929	6305	C00078
50	Uridine	15.54	217	HMDB0000296	6029	C00299
51	Serotonin	15.77	174	HMDB0000259	5202	C00780
52	5-Methylthioadenosine	17.5	236	HMDB0001173	439176	C00170

[1)] HMDB, Human Metabolome Database; HMDB is a comprehensive, high-quality, freely accessible, online database of small molecule metabolites found in the human body.

[2)] PubChem; PubChem is a database of chemical molecules and their activities against biological assays.

[3)] KEGG; Kyoto Encyclopedia of Genes and Genomes. KEGG is a collection of databases dealing with genomes, biological pathways, diseases, drugs, and chemical substances.

Table 2. Significantly changed metabolites in melanoma cells after 48 h of incubation.

Metabolites	VIP score	48 h / 1 h (↑ / ↓)[1)]	*p* value	*q* value[2)]	Significance[3)]
Glycine	1.35	↑	3.56E-06	0.006	***
Xylonic acid	1.34	↑	1.01E-08	0.001	***
Inositol	1.34	↑	2.00E-05	0.010	***
Phosphoric acid	1.34	↑	5.55E-08	0.003	***
Citric acid	1.34	↑	4.14E-04	0.024	***
Oxaloacetic acid	1.34	↑	1.31E-04	0.016	***
3-Hydroxybutyric acid	1.34	↑	1.35E-07	0.004	***
Adenine	1.32	↑	1.41E-04	0.017	***
Tryptophan	1.31	↑	3.97E-04	0.022	***
Elaidic acid	1.31	↑	6.23E-06	0.007	***
Lysine	1.30	↑	1.45E-05	0.008	***
Phenylalanine	1.29	↑	4.16E-04	0.024	***
Glycolic acid	1.29	↑	8.77E-04	0.026	***
Lactic acid	1.29	↑	8.81E-04	0.028	***
Anthranilic acid	1.27	↑	4.93E-05	0.011	***
Ribose	1.27	↑	5.38E-05	0.013	***
Cystathionine	1.27	↑	6.34E-05	0.014	***
Methionine	1.26	↑	8.10E-05	0.015	***
Taurine	1.25	↑	1.43E-04	0.018	***
Alanine	1.24	↑	2.40E-04	0.019	***
Threonine	1.23	↑	3.22E-03	0.036	**
Fructose	1.22	↑	3.40E-04	0.021	***
5-Methylthioadenosine	1.21	↑	5.69E-04	0.025	***
Creatinine	1.19	↑	3.86E-03	0.039	**
Isoleucine	1.19	↑	4.02E-03	0.040	**
Malonic acid	1.16	↑	1.60E-03	0.029	**
Pyroglutamic acid	1.16	↑	6.83E-03	0.043	**
Malic acid	1.15	↓	1.84E-03	0.031	**
Hypotaurine	1.12	↑	2.92E-03	0.035	**
Gallic acid	1.12	↑	3.73E-03	0.038	**
Glutamine	1.11	↓	1.70E-02	0.050	*
Proline	1.10	↑	1.62E-02	0.049	*
Leucine	1.09	↑	1.24E-02	0.046	*
Mandelic acid	1.09	↑	5.31E-03	0.042	**
Glutamic acid	1.03	↓	1.11E-02	0.044	*
Aspartic acid	1.02	↓	1.37E-02	0.047	*

[1)] The vertical arrows (↓ and ↑) represent a decrease or increase in metabolite levels after incubation (48 hour).
[2)] False discovery rate (FDR); the false discovery rate of 5% was applied to all tests to correct for multiple testing.
[3)] Symbols (*) indicate significant difference (*, $p < 0.05$; **, $p < 0.01$; ***, $p < 0.001$).

Table 3. Significantly changed metabolites in melanoma cells treated with α-MSH after 48 h of incubation.

**Metabolites**	**VIP score**	**48 h / 1 h (↑ / ↓)** [1)]	***p* value**	***q* value** [2)]	**Significance**[3)]
Citric acid	1.34	↑	1.22E-10	0.001	***
Inositol	1.33	↑	4.09E-10	0.001	***
Glycine	1.33	↑	5.04E-06	0.011	***
Lactic acid	1.33	↑	2.76E-09	0.003	***
Oxaloacetic acid	1.32	↑	4.70E-08	0.006	***
Glycolic acid	1.32	↑	6.19E-09	0.004	***
Xylonic acid	1.31	↑	1.82E-06	0.010	***
Phosphoric acid	1.31	↑	4.61E-07	0.006	***
Adenine	1.31	↑	2.35E-07	0.005	***
3-Hydroxybutyric acid	1.31	↑	9.04E-05	0.018	***
Glutamine	1.31	↓	6.97E-07	0.008	***
Tryptophan	1.31	↑	7.25E-07	0.009	***
Phenylalanine	1.27	↑	9.40E-04	0.031	***
Fructose	1.27	↑	2.68E-05	0.013	***
Sorbitol	1.26	↑	3.82E-05	0.014	***
Lysine	1.25	↑	5.75E-05	0.015	***
Elaidic acid	1.25	↑	7.68E-05	0.016	***
Pyroglutamic acid	1.24	↑	1.04E-04	0.019	***
Taurine	1.24	↑	1.05E-04	0.020	***
Ribose	1.24	↑	1.12E-04	0.021	***
Alanine	1.23	↑	1.37E-04	0.023	***
Anthranilic acid	1.22	↑	9.73E-04	0.033	***
Methionine	1.21	↑	2.64E-03	0.040	**
5-Methylthioadenosine	1.21	↑	2.68E-04	0.024	***
Mandelic acid	1.21	↑	3.27E-04	0.025	***
Creatinine	1.20	↑	5.52E-04	0.029	***
Gallic acid	1.20	↑	4.13E-04	0.026	***
Threonine	1.19	↑	4.71E-04	0.028	***
Glucose	1.19	↓	3.64E-03	0.044	**
Glutamic acid	1.18	↓	6.44E-04	0.030	***
Malonic acid	1.16	↑	1.10E-03	0.034	**
Isoleucine	1.16	↑	1.15E-03	0.035	**
Cystathionine	1.14	↑	1.59E-03	0.036	**
Hypotaurine	1.14	↑	1.78E-03	0.038	**
Glycolic acid	1.13	↑	6.06E-03	0.045	**
Aspartic acid	1.11	↓	2.62E-03	0.039	**
Proline	1.11	↑	2.77E-03	0.041	**
Leucine	1.10	↑	3.16E-03	0.043	**
Fumaric acid	1.05	↑	7.06E-03	0.046	**
Malic acid	1.05	↑	7.17E-03	0.048	**
Glycerol	1.02	↑	1.04E-02	0.049	*

[1)] The vertical arrows (↓ and ↑) represent a decrease or increase in metabolite levels after incubation (48 hour).
[2)] False discovery rate (FDR); the false discovery rate of 5% was applied to all tests to correct for multiple testing.
[3)] Symbols (*) indicate significant difference (*, $p < 0.05$; **, $p < 0.01$; ***, $p < 0.001$).

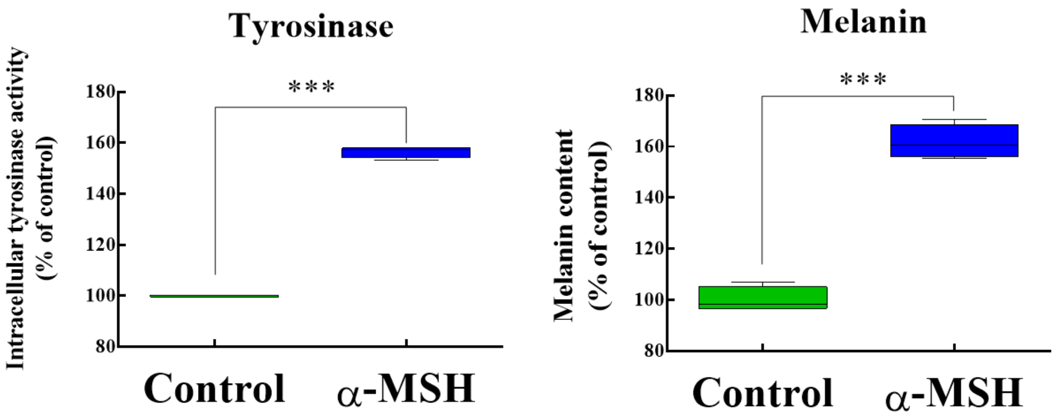

Figure 1. Box plots of tyrosinase activity and melanin content. Symbols (*) indicate significant difference (* $p < 0.05$; ** $p < 0.01$; *** $p < 0.001$).

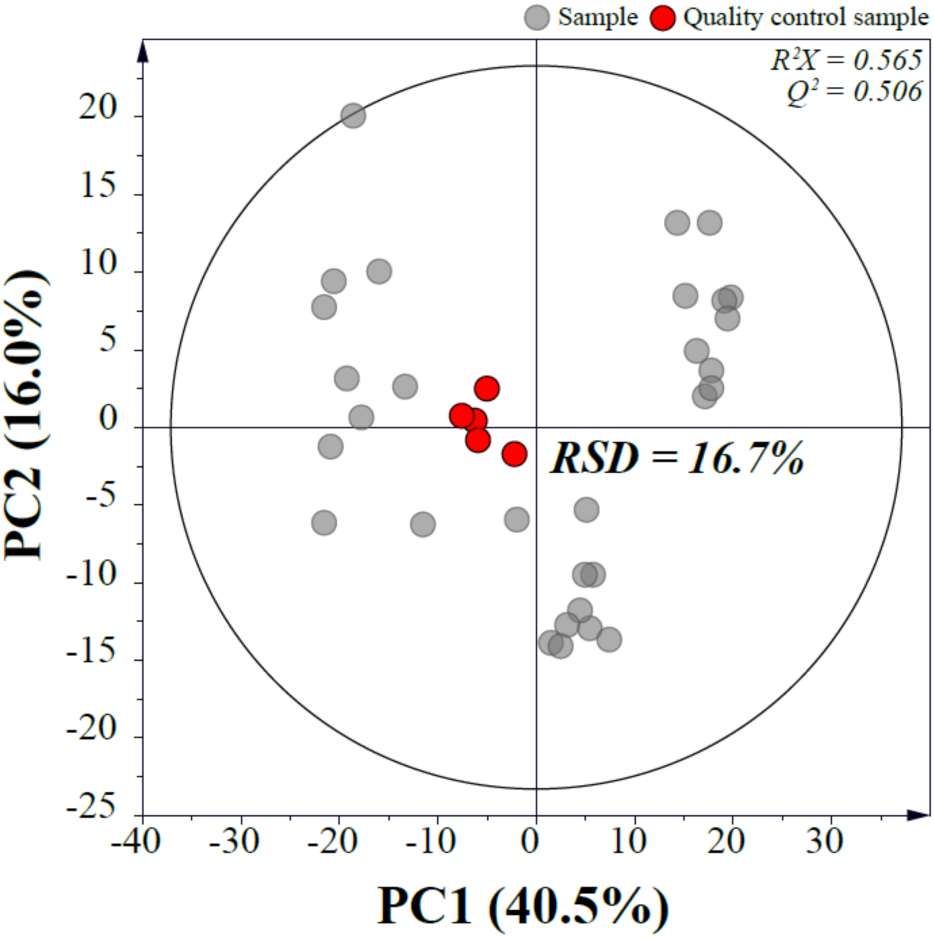

Figure 2. PCA score plot derived from GC–MS data of experimental samples (grey) and QC samples (red). QC samples were clustered centrally in the PCA score plot, ensuring the reliability of metabolomics analysis.
